# Resolution of Internal Carotid Dissection with Middle Cerebral Artery Occlusion in Pregnancy

**DOI:** 10.1155/2015/398261

**Published:** 2015-03-30

**Authors:** Nicole Ulrich, Amanda Johnson, Dominique Jodry, Chi Dola, Sheryl Martin-Schild, Ramy El Khoury

**Affiliations:** ^1^Stroke Program, Department of Neurology, Tulane University School of Medicine, New Orleans, LA 70112, USA; ^2^Section of Maternal-Fetal Medicine, Department of Obstetrics and Gynecology, Tulane University School of Medicine, New Orleans, LA 70112, USA

## Abstract

*Introduction*. Cervical artery dissection (CAD) is a common cause of stroke in younger patients. While the incidence of stroke in pregnancy is increasing, CAD remains a rare cause of ischemic stroke in the pregnant population, with only 30 cases described in the literature, most in the postpartum period. *Methods*. The case of a pregnant patient at 18 weeks of gestation presenting with CAD and ischemic stroke following intercourse is discussed. *Discussion*. CAD results from an intimal tear in the carotid artery, allowing accumulation of blood in the vessel wall. Stroke results from embolization of thrombogenic material in the wall. Etiology includes minor trauma, connective tissue disorders, or anatomic variations of the carotid artery. Most patients present with headache and/or neck pain, while ischemic symptoms are seen in at least 50% of patients. In the pregnant population, imaging with MRI or MRA of the head and neck aids in diagnosis. Once the diagnosis is made, patients are treated with either anticoagulation or antiplatelet medications. The optimal treatment in both pregnant and nonpregnant patients has not been well-studied. *Conclusion*. CAD is an important diagnosis to consider in a pregnant patient with persistent headache, especially if neurological symptoms are present. Imaging should be quickly obtained so treatment can be initiated.

## 1. Introduction

Stroke is recognized as an important contributor to maternal morbidity and mortality in pregnancy, with the rate of 1 in 6000 deliveries, contributing to 12% of maternal deaths [1]. It carries a major risk of long-term disability and death. The incidence of stroke in pregnancy and the postpartum period is estimated at 4 to 40 strokes per 100,000 pregnancies, and it is increasing, likely the result of an ageing population [2, 3]. With improved noninvasive imaging techniques, internal cervical artery dissection (CAD) is increasingly recognized as a common cause of stroke in younger patients [4, 5]. While the overall incidence is 2.6 per 100,000 people, CAD accounts for up to 20 percent of strokes in younger patients, with a mean age of 40 to 46 years [5, 6]. CAD remains a rare etiology of stroke in pregnancy [7, 8]. In this paper, we present a case of CAD with associated ischemic stroke in pregnancy.

## 2. Case Report

A 35-year-old African American female, gravida 2 para 0-1-0-0 presented to the hospital at 18 weeks of gestation with left-sided upper and lower extremity weakness. This weakness began suddenly after intercourse with her husband three hours prior to presentation, lasted for about 30 minutes, and resolved. Associated symptoms included tingling in her left arm and leg, slurred speech, and onset of a right retro-orbital headache with minimal photophobia approximately 60 minutes after onset of the weakness. The patient's medical history was significant for hypertension, controlled with hydrochlorothiazide, and complicated migraine. Her previous pregnancy 6 years earlier was significant for development of thrombotic thrombocytopenic purpura (TTP) at 22 weeks of gestation and complicated by bacterial endocarditis, left hip osteomyelitis, and subacute left hemispheric stroke. No imaging studies from that time are available, but computed tomography (CT) scan performed approximately 5 years after the subacute left hemispheric stroke showed no residual defects from this stroke.

**Figure 1:**
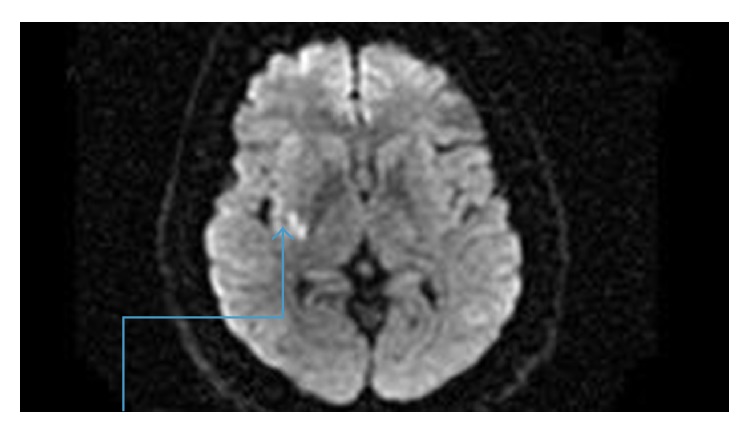
Multifocal infarction in the right middle cerebral artery territory noted upon admission to the hospital.

**Figure 2:**
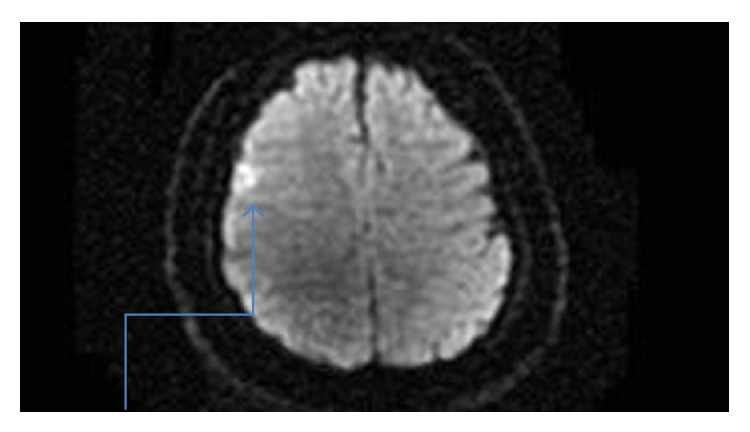
Another multifocal infarction within the middle cerebral artery territory was noted upon admission.

**Figure 3:**
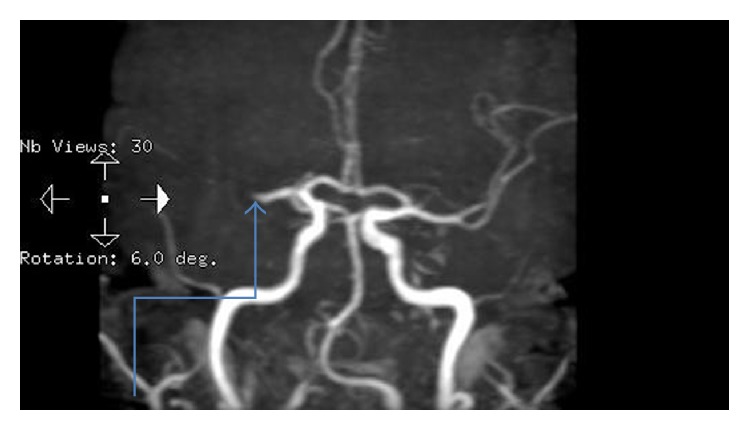
Right middle cerebral artery occlusion was detected upon admission.

**Figure 4:**
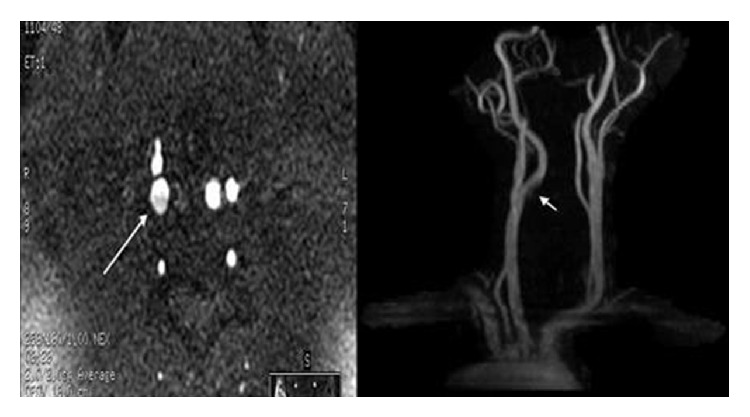
Dissected right internal carotid artery was detected upon admission.

At the time of presentation, her vital signs were within normal limits. Neurologic exam was significant for mild left facial flattening and drooping. Sensation, motor strength in both upper and lower extremities, coordination, and reflexes were all within normal limits. CBC was significant for a hematocrit of 33.6% and platelets of 154,000/microL. Comprehensive metabolic profile and coagulation studies were within normal limits. An ADAMTS 13 (Disintegrin and Metalloprotease with a ThromboSpondin type 1 motif, member 13) activity from one week earlier decreased to 16% of normal. Echocardiography was normal. Magnetic resonance imaging (MRI) and magnetic resonance angiography (MRA) of the head and neck without contrast were performed. These imaging studies revealed a restriction correlating with an acute ischemic event in the right middle cerebral artery (MCA) territory, occlusion of the right MCA just distal to the Circle of Willis, and a right internal CAD at the level of the ostium with irregularity of the lumen (Figures 1, 2, 3, and 4). Magnetic resonance venography (MRV) was negative.

**Figure 5:**
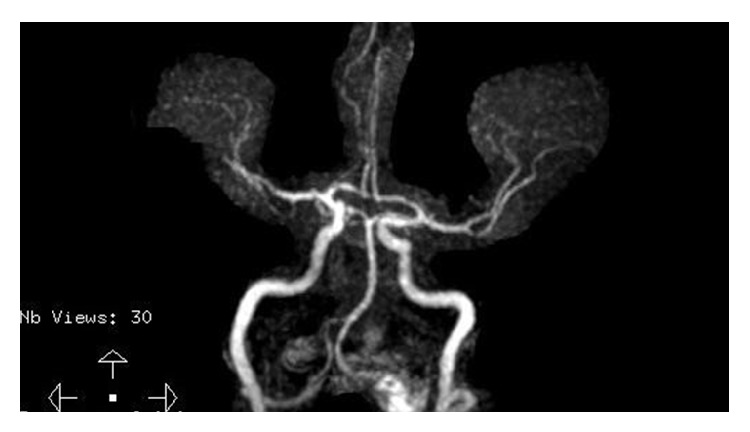
The middle cerebral artery was noted to have recanalized when repeat MRI studies were performed at just beyond 6 weeks postpartum.

**Figure 6:**
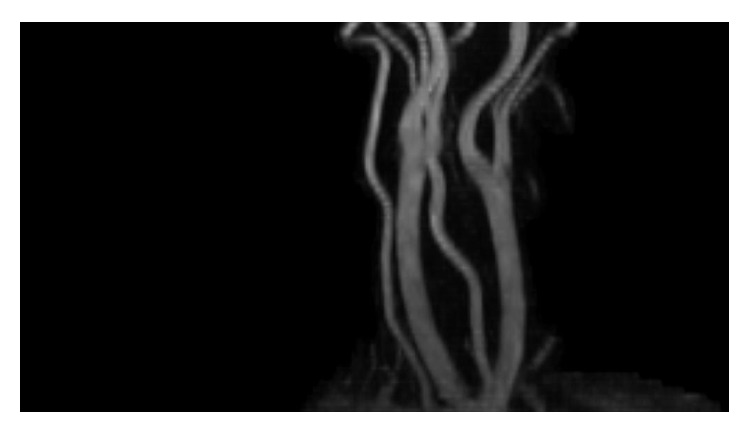
The previously dissected internal carotid artery was now well healed at just beyond 6 weeks postpartum.

The patient was admitted to the hospital and anticoagulation was initiated with enoxaparin. Throughout her hospital stay, the patient exhibited no further neurologic deficits. Her facial flattening and drooping resolved. Her NIHSS stroke scale remained at zero. She was discharged home on hospital day 4. Anticoagulation therapy with therapeutic dosing of enoxaparin was continued throughout pregnancy until 6 weeks postpartum. Pregnancy was complicated by the development of gestational diabetes and superimposed preeclampsia, and the patient delivered a viable infant at term. Six weeks after delivery, MRI and MRA of the head and neck were repeated. MRI revealed evidence of a remote insult. MRA revealed a patent right MCA and no visible CAD (Figures 5 and 6).

## 3. Discussion

CAD results from an intimal tear, with blood penetrating through the tear and accumulating in the wall. In the carotid artery, the tear often develops about 2 cm distal to the carotid bulb and then extends distally [5, 6]. If a hematoma develops, luminal narrowing or even occlusion of the artery occurs [5, 6]. Cerebral ischemia associated with CAD is thought to result from artery-to-artery embolization from release of the thrombogenic material in the arterial wall [6, 9]. Less commonly, it may result from hemodynamic compromise of brain perfusion secondary to narrowing of the carotid artery [6].

While spontaneous cases do occur, the etiology of CAD is often thought to be minor head or neck trauma, structural changes in the artery such as connective tissue disorders, or anatomic variations [6, 10]. Examples of trauma include a blunt trauma to the head, abrupt turning of the neck, violent coughing or vomiting, chiropractic manipulation of the neck, and rhythmic flexion and extension of the head, such as with head banging [5, 6]. Medical conditions associated with an increased risk of CAD include migraine, which has a twofold increase, hyperhomocysteinemia, recent systemic infections, and connective tissue disorders [11, 12]. In our patient, we hypothesize that the CAD was precipitated by sexual intercourse, as the symptoms began during this activity.

Most patients with CAD will present with headache and/or neck pain [5, 6]. Approximately half of cases of CAD will also demonstrate ipsilateral Horner's syndrome as a result of compression of sympathetic nerve fibers in the vessel wall [6]. Finally, 50 to 90% of patients may present with signs of cerebral or retinal ischemia, resulting in symptoms of amaurosis fugax, TIA, or stroke. These symptoms may be delayed, presenting up to one month after the dissection [2, 5]. In the nonpregnant population, CAD has a mortality of less than 5%. Seventy-five percent of patients have a favorable functional outcome and 46–90% experience resolution of stenosis [13].

The incidence of stroke in pregnancy has been increasing, especially with higher rates of obesity, hypertension, and cardiac disease, in addition to pregnancy-related complications of preeclampsia and a hypercoagulable state. The risk of stroke associated with pregnancy is especially increased in the peripartum period, a time of great physiologic change [14]. Although CAD is a common cause of stroke in young, nonpregnant patients, CAD remains a relatively rare cause of stroke in pregnant patients, accounting for only 6% of dissections in women younger than 50 years [15]. Previous literature has documented 27 cases in the postpartum period, but only three other cases in the antepartum period [7, 12, 15, 16].

Pregnancy presents its own unique circumstances that may increase the risk of CAD. Vascular wall remodeling that results in increased vascular volume and compliance may make vessels more susceptible to dissection, while increased cardiac output and blood flow through the carotid arteries may lead to changes in shearing forces across the endothelial surface, resulting in greater risk for an intimal tear [11, 17, 18]. The hypercoagulable state of pregnancy contributes to development of thrombosis at the area of dissection, with subsequent development of embolism [11, 17]. In addition, expulsive efforts in the second stage of labor may theoretically precipitate dissection [11, 15, 18].

As with the nonpregnant population, the main presenting symptoms of CAD in pregnant women are headache and neck pain [15]. A potential for delay in diagnosis exists secondary to the high prevalence of headache in pregnancy and the postpartum period. Other common causes of headache in pregnancy and the postpartum period include preeclampsia/eclampsia, postdural puncture headache, caffeine withdrawal, sinusitis, and lactation headache [11]. However, in patients with a persistent headache and associated neurological symptoms, CAD must be considered in the differential diagnosis, with appropriate imaging performed to evaluate the presence of a dissection and initiate appropriate treatment if present [11, 15].

In addition to stroke, our patient's history necessitates that a relapse of TTP must be considered in her differential diagnosis. Previous case reports have demonstrated that despite no findings of thrombocytopenia or microangiopathic hemolytic anemia on CBC, a patient presenting with stroke-like symptoms may be experiencing an atypical presentation of TTP rather than a thromboembolic stroke [19]. To differentiate between the two conditions, ADAMTS13 plasma activity can be measured. ADAMTS13 is responsible for cleaving unsually large von Willebrand factor (ULVWf) multimers into their normal circulating forms. A mutation in the ADAMTS 13 gene leads to reduced ADAMTS13 cleaving activity, resulting in accumulation of ULVWf multimers, platelet aggregation, and platelet thrombi associated with TTP. Thus, a low level of ADAMTS13 would be more suggestive of TTP as a cause of symptoms [19].

Noninvasive MRI, MRA, and CTA are emerging as the new standard in CAD diagnosis, though catheter angiography remains the gold standard [4, 13]. Due to our patient's pregnant state, we used MRI and MRA for diagnosis. Intramural hematoma, with typical time related signal change, is the most common finding of CAD seen on MRI [4]. Contrast enhanced MRA using gadolinium offers faster acquisition time, is less susceptible to artifact than noncontrast studies, and provides visualization of the vessels regardless of flow or tortuosity [13]. However, noncontrast may be preferred for lower cost and utility in patients for whom gadolinium is not recommended, as in our patient [13]. Gadolinium has a teratogenic effect in pregnant animals and is considered category C in pregnancy by the FDA [20]. Routine use is not recommended and ACR guidelines allow its use in pregnant patients only when the provider has engaged in a well-documented risk-benefit analysis [21].

Treatment of CAD often includes use of anticoagulation or antiplatelet therapies. Anticoagulation is used primarily to prevent embolization from the dissected artery to more distal sites. Theoretical risks of use of anticoagulation include hemorrhagic transformation of cerebral infarct or hematoma formation at the site of dissection [5]. Anticoagulation is usually initiated with heparin and transitioned to warfarin, often for a total of 3 months. At this time, imaging is repeated. If the arterial abnormality has resolved, patient is transitioned to antiplatelet therapy. If abnormalities remain, anticoagulation may be continued for another 3 months, with repeat imaging at that time [5, 6]. If anticoagulation cannot be used, antiplatelet therapy, such as aspirin, is an acceptable alternative treatment. A Cochrane review of available observational studies and nonrandomized trials showed no significant difference between the two therapeutic options [22]. Recently, the results of the cervical artery dissection (CADISS) trial were published [23]. This trial included 250 patients with extracranial carotid or vertebral artery dissection who, within seven days of symptom onset, were randomly assigned to treatment with antiplatelet therapy or anticoagulation therapy for three months. The choice of the antiplatelet or anticoagulation drug used was left to the discretion of the treating physician. The trial found no significant difference in the recurrence of stroke at three months after the initial event. Only four cases (2% of patients) of ipsilateral stroke occurred in the study period, with three in the antiplatelet group and one in the anticoagulant group. The stroke in the anticoagulant group was also significant because it was hemorrhagic in nature. Overall, the rate of recurrence was low, and lower than previous observational studies demonstrated, and thus a definitive recommendation of antiplatelet or anticoagulation therapy could not be made [23]. Surgical treatment is reserved for those patients with continued ischemic symptoms despite medical therapy or progressing aneurysms [6].

As with the nonpregnant population, optimal treatment for pregnant patients with CAD has not been determined. While postpartum patients may be treated with either anticoagulation or antiplatelet agents as described above, pregnancy limits the options available. Anticoagulation treatment is limited to use of heparin or low molecular weight heparin (LMWH) in pregnancy, which are both considered safe to use in pregnancy. Warfarin is contraindicated in this population due to the risk of adverse fetal effects with use of the medication. The American Congress of Obstetricians and Gynecologists (ACOG) recommends use of therapeutic doses of anticoagulation in cases of thromboembolism in pregnancy. Anticoagulation is then continued for at least 6 weeks in the postpartum period, a time of high risk for thromboembolic events [24]. If the treatment may be discontinued after 6 weeks, often heparin or a low molecular weight heparin is used. However, if she will continue to require anticoagulation outside of the postpartum period, the patient may be transitioned to warfarin therapy. Heparin, LMWH, and warfarin are all compatible with breastfeeding [24].

With future pregnancy, the patient should be counseled about the recurrence risk with CAD. Up to 4% of patients with CAD will have recurrence of the event, with few risk factors for recurrence identified [10]. Thus far, no evidence is available to determine whether anticoagulation or antiplatelet therapy prevents recurrence [6]. Thus, recommendations for use of prophylactic anticoagulation with heparin or LMWH or use of antiplatelet agents, such as low-dose aspirin, with subsequent pregnancies cannot be definitively given.

## 4. Conclusion

It is important to consider CAD in a patient with neurologic symptoms associated with headache, neck pain, or orbital pain in both the antepartum and postpartum period, so that appropriate imaging and treatment can be undertaken.
